# Association between cardiovascular diseases and pregnancy-induced hypertensive disorders in a population of Cameroonian women at Yaoundé: A case-control study

**DOI:** 10.1371/journal.pone.0225591

**Published:** 2019-12-16

**Authors:** Benedict P. Taa Nguimbis Esseme, Ebongue Mbondji

**Affiliations:** 1 Department of Public Health, School of Health Sciences, Catholic University of Central Africa, Yaounde, Center Region, Cameroon; 2 Department of Health Systems and Policy, School of Health Systems and Public Health, University of Pretoria, Pretoria, Gauteng Province, South Africa Republic; University of Mississippi Medical Center, UNITED STATES

## Abstract

**Background:**

Positive associations have been found between Hypertensive Disorders of Pregnancy gestational hypertension, preeclampsia and cardiovascular diseases within non-African populations, no data exist from sub-Saharan Africa. We aimed to assess this association in Cameroonian mothers.

**Methods:**

We used a case-control study. Cases were women diagnosed with any arteriosclerotic cardiovascular disease between 2012 and 2017 at two major hospitals of Yaoundé. Controls were mothers of children who sought pediatric care at the Gyneco-obstetric hospital of Yaoundé, with no diagnosis of cardiovascular disease. We abstracted data from patient files to assess cardiovascular disease and used phone-based questionnaires to assess a prior history of Hypertensive Disorders of Pregnancy. We used logistic regression and propensity scores for data analysis.

**Results:**

Out of 1228 individuals selected, 173 cases and 339 controls participated in the study. We found no increased risk of cardiovascular diseases for women with a history of Hypertensive Disorders of Pregnancy (OR = 0.83, 95% CI, 0.51 to 1.34). Women with gestational hypertension had 2.33 (95% CI, 0.99 to 5.50) times the risk of women with no history of Hypertensive Disorders of Pregnancy, an inverse association was observed between preeclampsia and cardiovascular diseases (OR = 0.28, 95% CI, 0.10 to 0.72).

**Conclusions:**

Cameroonian women with a history of gestational hypertension may have a higher risk of cardiovascular diseases. However, population-based studies with more accurate data on the exposure are needed.

## Introduction

Hypertensive Disorders of Pregnancy (i.e. preeclampsia, gestational hypertension and eclampsia) are recognized factors of maternal morbidity and mortality. The World Health Organization (WHO) multicounty survey on maternal and newborn health, estimated the global cumulative incidence of Hypertensive Disorders of Pregnancy between 2004 and 2008 at 2.7% overall; with a cumulative incidence of 2.2% for preeclampsia, 0.3% for gestational hypertension and 0.3% for eclampsia [1]. In the African region, the cumulative incidence between 2002 and 2010 was much higher with 5.6% for preeclampsia, and 2.9% for eclampsia [2].

Previous studies have shown that these disorders during pregnancy negatively influence postpartum health outcomes. Williams (2003) [3], as well as Roberts and Hubel (2003) [4], highlighted Hypertensive Disorders of Pregnancy as essential determinants, that promote poor women’s cardiovascular health postpartum. Many studies have found a positive association between Hypertensive Disorders of Pregnancy and diagnosis of cardiovascular diseases later in life [5–8]. Most of these studies suggesting around 1.5 to 2-fold elevated risk. However, to the best of our knowledge, no such data exist for women from sub-Saharan Africa. We aimed to investigate whether a positive association between Hypertensive Disorders of Pregnancy and cardiovascular diseases exists in a population of Cameroonian women at two reference hospitals of Yaoundé.

## Methods

### Study setting

We recruited participants from the General Hospital and the Gyneco-obstetric and Pediatric hospital of Yaoundé, Cameroon. Both hospitals are located on the same site and share the same catchment area. The General Hospital of Yaoundé is the main cardiovascular disease treatment centre in Yaoundé, while the Gyneco-obstetric and Pediatric hospital of Yaoundé is the main health facility of maternal and pediatric care. Patients who seek pediatric consultations at the General hospital are usually referred to the Gyneco-obstetric and Pediatric hospital for admission, likewise, patients who seek cardiovascular consultation at the Gyneco-obstetric and Pediatric hospital, are referred to the General Hospital.

### Participants

We used a case-control design and considered patients with complete administrative data (date of birth, name, phone number, name of the mother, name and phone number of a relative), residing in Yaoundé during the study period (from the 1^st^.01.2012 to the 5^th^.12.2017). Eligible cases consisted of all mothers aged 18 to 60 years old who were diagnosed with any form of arteriosclerotic cardiovascular diseases at the General Hospital and the Gyneco-obstetric hospital during the study period. At the General Hospital, we only found the files of patients who continued to receive health care from the General hospital between 2015 to 2017; files of patients diagnosed with the cardiovascular disease between 2012 and 2014, who did not continue their cardiology consultations at the General hospital were missing.

Controls consisted of mothers of children admitted at the Gyneco-obstetric hospital during the same period where the cases were diagnosed. In the absence of a patient roster for admitted children, we associated the first letters of the last name of each child to the end digit of their hospital registration number. We randomly selected 10 letter-digit combinations from the patients letters-digit pool. We limited the selection to 10 arbitrary. We included all the patients who had the letter-digit combinations we selected.

We excluded: 1) participants with a diagnosis of any type of diabetes mellitus or any form of renal disease; 2) women with a diagnosis of cardiovascular diseases before their very first pregnancy; 3) women pregnant less than six months before the 5^th^ October 2017; 4) participants who neither spoke English nor French.

### Outcome assessment

We abstracted the patients’diagnosis from the hospital files and included patients with any coronary heart diseases (ICD codes I20 to I25.9), cerebrovascular diseases (I60 to I69.8), and hypertensive diseases (I10 to I15.9). When the diagnosis was absent from patients’ files, we relied on the patients’ drug prescription. We sought for the disease indicated for the treatment the physician prescribed and classified the patient accordingly. When the diagnosis and the drug prescription were missing, we used the recorded blood pressure. If the patients had a systolic blood pressure ≥ 140 mmHg or a diastolic blood pressure ≥ 90 mmHg during three successive visits to the cardiologist, we included the patients as having any form of hypertensive disease. We aggregated the cardiovascular disease diagnosis in a single dichotomous variable.

### Exposure assessment

We assessed the history of Hypertensive Disorders of Pregnancy among participants using phone-based interviews. We used the standardized questionnaire from Diehl et al (2008) [9] which achieved an 80% sensitivity and a 90% specificity in a group of women with a greater than 20-year history of preeclampsia. We translated the questionnaire into French and pretested it at the General Hospital of Yaoundé, for better wording. We trained five medical personnel to conduct interviews with the participants. Prior to the interviews, we sent text messages to all the participants to inform them about the study. When participants were not answering, we called them back during two successive days three times per day. When the contact number on the file was a relative of the participant, we asked the participant’s phone number to the relative. We also set appointments for phone interviews when the patient asked to be called later. We conducted all the interviews either in French or in English, from the 13^th^ December 2017 to 23rd April 2018.

We defined Hypertensive Disorders of Pregnancy to be present if a participant answered positively to any of the questions a-c below:

a During any of your pregnancies which lasted for more than 5 months or 20 weeks, which of the following did the physician diagnose? 1. Only high blood pressure or hypertension 2. Pre-eclampsia 3. Eclampsia 4. None of these diseases
b During any of your pregnancies, which lasted for more than five months or 20 weeks, did a physician ever tell you that you had high blood pressure or hypertension?
c During any of your pregnancies which lasted for more than five months or 20 weeks, were you prescribed any drug to lower your blood pressure or taking drugs such as Adalate, Aldomet, Loxen, or Tradate?

We classified as Preeclamptic/Eclamptic women who responded positively to any of the previous questions and positively to any of the d and e questions below. We considered individuals to have gestational hypertension if they answered positively to at least one of a to c questions but responded negatively to d and e of the following questions. Participants who responded negatively to both sets of questions were considered as not having a history of any type of hypertensive disorders of pregnancy.

d Did your doctor tell you that, you had Protein in the urine or any positive urine test during any of the pregnancies where you had a diagnosis of high blood pressure, or given a drug to lower your blood pressure?
e During any of the pregnancies where you had a diagnosis of high blood pressure or given a drug to lower your blood pressure, did you have any seizure or loss of consciousness?

### Sample size

We planned a study with one control per case. Prior data suggest that the odds ratio between a history of Hypertensive Disorders of Pregnancy and coronary heart diseases was 2.28 [10] if the prevalence of Hypertensive Disorders of Pregnancy among African women is 5.6% [2]. We needed 220 cases and 880 controls to achieve an 82% power to reject the null hypothesis at a 95% confidence level. However since we wanted to anticipate for unreachable phone calls and the loss of patients’files in the registration services of both hospitals, we included all the eligible case and twice the number of cases for controls.

### Statistical analyses

We compared the distribution of Hypertensive Disorders of Pregnancy (i.e. as an aggregate variable, Gestational hypertension, and Pre-eclampsia) among cases and controls.

We fitted four crude and adjusted logistic regression models to assess the association between Hypertensive Disorders of Pregnancy and cardiovascular diseases.

In the first model, we evaluated the association between any Hypertensive Disorder (i.e. either gestational hypertension or preeclampsia) of Pregnancy and cardiovascular disease. We then analyzed the association between each type of Hypertensive Disorders of Pregnancy and cardiovascular disease: Model 2: women with preeclampsia (E_1_) vs women with no history of hypertensive disorder of pregnancy (E_0_). Model 3: women with gestation hypertension (E_2_ vs E_0_). Model 4 treated Hypertensive Disorders of Pregnancy like a polytomous variable consisting of E_0_, E_1_, and E_2_, the reference level was E_0_.

For the adjusted analysis we considered the following covariates: smoking status (current, former smoker, never smoker), participant’s age (age was included as a continuous variable since a test for linearity yielded non significant results), multiple gestation (yes/no), family history of cardiovascular diseases (yes/no), total number of pregnancies (<2, 2, >2; the cutoff was chosen since a previous study suggested a reduced risk of cardiovascular disease for 2 or more pregnancies [4]), marital status (single, married or unmarried couple, widowed, divorced) and level of education (less than secondary, secondary, higher than secondary). We computed propensity scores for each patient, which was the probability of each participant to have Hypertensive Disorders of Pregnancy given the covariates of the adjusted analysis. We categorized the propensity scores in six classes (default setting) and included these classes in a logistic regression model for models 1 to 3. For model four we used a classical multivariable logistic regression with the same covariates. We presented the resulting odds ratio (crude and adjusted), and 95% confidence intervals.

### Sensitivity analyses

We performed sensitivity analyses using Greenland (2006) [11] methods to assess the impact of exposure misclassification on the binary Hypertensive Disorder of Pregnancy variable. We considered that misclassification was either independent nondifferential or independent differential. We performed the analysis on the strata of propensity scores and used the Mantel-Haenzel formula to pool the estimates. We obtained sensitivity and specificity estimates from Klemmensen et al. (2007) [12] and Diehl et al. (2008) [9]. We computed the E-value [13] to assess how strong unmeasured confounders would need to be to explain away the observed exposure-outcome relationship. We computed E-values on the adjusted estimates, under the assumption of low outcome incidence.

### Ethical considerations

We obtained oral informed consent from each participant before beginning the interviews. We read the consent form to each participant before every interview. We started the interview if and only if the participant verbally agreed with the terms in the consent form. We could not obtain written consent as none of the participants had their postal code recorded. The Catholic University of Central Africa and the ethical review committee of the Gyneco-obstetric and Pediatric hospital of Yaoundé approved the study protocol, and the way we planned to obtain consent from participants. Both hospitals provided anonymized patients files.

## Results

**Fig 1:**
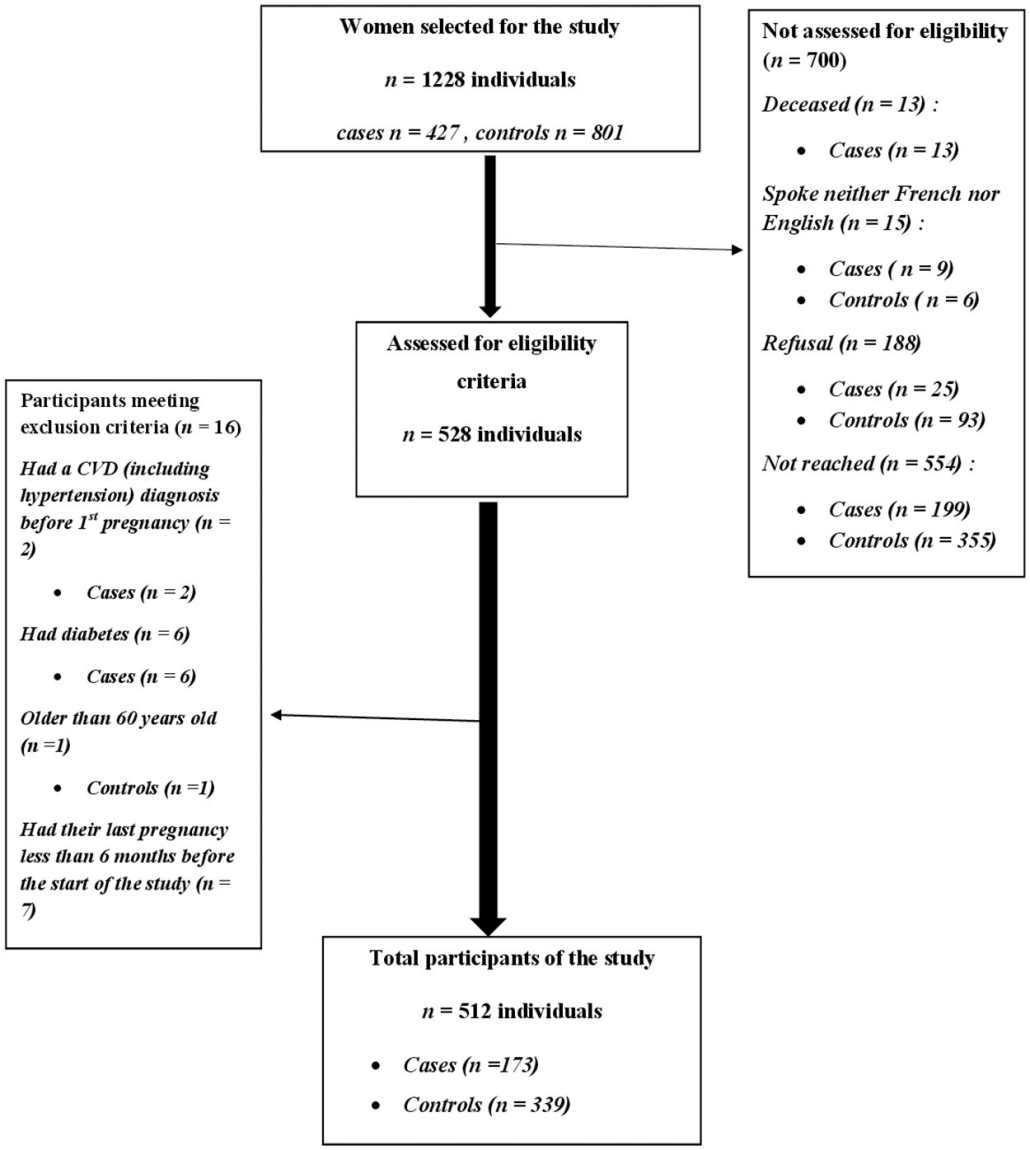
Participation flow diagram.

From the 1228 participants (427 cases and 801 controls) selected, we did not reach 199 (46.6%) cases, and 355 (44.3%) controls by phone. Overall 6% (n = 25) of cases, and 11.6% (n = 93) of controls refused to participate; 2% (n = 9) of cases and <1% (n = 6) of controls neither spoke French nor English, 3% (n = 13) of cases have died, and 2% (n = 8) of the cases, 1% (n = 7) of the controls met exclusion criteria. Fig 1 presents the participation at each stage of the study.

Table 1 summarizes the participants’ characteristics. Cases were older and had more pregnancies than controls (5 versus 3). Controls had higher educational attainment, 37% of controls compared to 19.4% of cases reported attending a higher education institution. About 17% of cases and around 2% of controls reported being a widow. Controls had a higher proportion of singles than cases (25.4% vs 9.4%), which is consistent with the latest DHS Cameroon survey published in 2011 [14], suggest that 26% of households heads were women, among these households, around 60% of these households were single parent. We also noted a lower proportion of divorced among controls than cases (<1% vs 6%). We did not find any difference among cases and controls in terms of family history of pregnancy, smoking status and multiple pregnancies.

**Table 1:** Characteristics of study participants.

Variables	Cases (*n* = 173)	Controls (*n* = 339)	Total (*n* = 512)	P value
**Median age at diagnosis (IQR**^b^**)**	49 (11.75)	29 (9)	34 (20)	<0.001
Missing observations	3	21	24	
**Median number of pregnancies (IQR)**	5 (3)	3 (2)	3 (3)	<0.001
Missing observations	3	2	5	
**Multiple pregnancy**				0.212
Yes	24 (14%)	63 (18.8%)	87 (17.2%)	
No	148 (86%)	272 (81.2%)	420 (82.8%)	
Missing observations	1	4	5	
**Smoking status**				0.377
Current smokers	3 (1.76%)	10 (3.02%)	13 (2.59%)	
Former smokers	17 (10%)	23 (6.95%)	40 (7.98%)	
Never smokers	150 (88.2%)	298 (90%)	448 (89.4%)	
Missing observations	3	8	11	
**Family history of CVDs**				0.634
Yes	82 (48.2%)	154 (45.6%)	236 (46.5%)	
No	88 (51.8%)	184 (54.4%)	272 (53.5%)	
Missing observations	3	1	4	
**Education attainment**				<0.001
Less than secondary	49 (28.8%)	36 (10.9%)	85 (17%)	
Secondary	88 (51.8%)	172 (52.1%)	260 (52%)	
Higher education	33 (19.4%)	122 (37%)	155 (31%)	
Missing observations	3	9	12	
**Marital status**				<0.001
Divorced	10 (5.88%)	3 (0.89%)	13 (2.57%)	
Couple (Married & Unmarried)	115 (67.6%)	242 (72.2%)	357 (70.7%)	
Single	16 (9.41%)	85 (25.4%)	101 (20%)	
Widow	29 (17.1%)	5 (1.49%)	34 (6.73%)	
Missing observations	3	4	7	

^b^ interquartile range

We summarized the prevalence of a self-reported history of Hypertensive Disorders of Pregnancy in Table 2. The reported prevalence of Hypertensive Disorders of Pregnancy was similar in cases (20.3%) and controls (21.8%). However, the proportion of women who reported having a history of gestational hypertension was higher in cases than in controls (11.6% vs 8%). Cases had a slightly lower history of preeclampsia than controls (8.7% vs 12.8%).

**Table 2:** Distribution of the exposure among cases and controls.

Variable	Cases	Controls	Total	P value
**Hypertensive disorders of pregnancy** *				0.80
No	137 (79.7%)	248 (78.2%)	385(78.7%)	
Yes	35 (20.3%)	69 (21.8%)	104 (21.3%)	
Missing observations	1	22	23	
**Hypertensive disorders of pregnancy** **				0.20
No	137 (79.7%)	248 (79.2%)	385 (79.4%)	
Gestational hypertension	20 (11.6%)	25 (7.99%)	45 (9.28%)	
Preeclampsia	15 (8.72%)	40 (12.8%)	55 (11.3%)	
Missing observations	1	26	27	

* Hypertensive Disorders of Pregnancy treated as a dichotomous variable with two outcomes.

** Hypertensive Disorders of Pregnancy treated as a polytomous variable with three possible outcomes.

We report the results of the crude and adjusted analysis in Table 3. There was no evidence for an association of Hypertensive Disorders of Pregnancy and cardiovascular diseases (adjusted OR = 0.83, 95% CI: 0.51 to 1.45). Similarly, gestational hypertension was not associated with cardiovascular disease (adjusted OR = 1.47, 95% CI: 0.77 to 2.79). However, we found an inverse association between pre-eclampsia and cardiovascular diseases (adjusted OR = 0.47, 95% CI: 0.22 to 0.89).

**Table 3:** Results of the Crude and adjusted analyses.

Exposure	Unadjusted results Odds ratio (95% CI)	Adjusted results Odds ratio (95% CI)	E-value
**Hypertensive disorder of pregnancies**^a^	n = 489	n = 448	1.70
	0.92 (0.58 to 1.45)	0.83 (0.51 to 1.34)	
**Gestational hypertension**^b^	n = 395	n = 395	2.3
	1.37 (0.73 to 2.57)	1.47 (0.77 to 2.79)	
**Pre-eclampsia**^c^	n = 403	n = 403	3.87
	0.61 (0.32 to 1.18)	0.45 (0.22 to 0.89)	
**Hypertensive Disorders of Pregnancy**^d^	n = 385	n = 321	
No HDP history (ref)	1	1	
Gestational hypertension	1.45 (0.78 to 2.7)	2.33 (0.99 to 5.50)	4.09
Preeclampsia	0.68 (0.36 to 1.27)	0.28 (0.10 to 0.72)	6.60

^a,b,c^ we computed propensity scores and categorized them in six classes (default setting), we later added them to a logistic regression model with a dichotomous exposure.

^d^ we used a classical multiple regression model to adjust for confounding, we treated the exposure was polytomous with three levels.

We adjusted for smoking status, age, multiple gestations, family history of cardiovascular diseases, number of pregnancies, marital status, and level of education.

When we treated Hypertensive Disorders of Pregnancy as a polytomous variable (absent, gestational hypertension, preeclampsia, [model 4]), we found a positive association between gestational hypertension and cardiovascular diseases (Adjusted OR = 2.33, CI: 0.99 to 5.50). Similar to when treated as a dichotomous variable, we found an inverse association between preeclampsia and cardiovascular diseases (adjusted OR = 0.28, CI: 0.10 to 0.72). E-values were higher in the polytomous model as compared to the corresponding dichotomous models. Among the estimates in the different dichotomous adjusted models, the E-value was the highest for pre-eclampsia (E-value = 3.87) and the lowest for the Hypertensive Disorders of Pregnancy (Yes/No) model (E-value = 1.7).

Table 4 displays the results of the sensitivity analysis for exposure misclassification by increasing specificity of the aggregate Hypertensive Disorders of Pregnancy variable. Under non-differential misclassification, all the corrected estimates are further away from the null than the uncorrected adjusted estimate (OR = 0.83), they increased as the specificity increased to come closer to the null. We did not observe a similar pattern for increasing or decreasing estimates of sensitivity.

**Table 4:** Estimates for the sensitivity analysis of the exposure (presented by increasing specificity).

Cases		Controls						
Sens.*	Spe**.	Sens.*	**80**	**85.04**	**58.46**	**98**	**72.58**	**27.42**
		Spe.**	**90**	**92.22**	**92.64**	**95**	**98.62**	**99.69**
**80**	**90**		0.59^d^	0.51	0.32	0.50	0.29	0.1
**85.04**	**92.22**		0.78	0.68^d^	0.43	0.64	0.38	0.13
**58.46**	**92.64**		1.25	1.08	0.69^d^	1.03	0.62	0.22
**98**	**95**		0.90	0.78	0.49	0.74^d^	0.45	0.15
**72.58**	**98.62**		1.61	1.40	0.89	1.33	0.80^d^	0.28
**27.42**	**99.69**		4.61	4.00	2.54	3.80	2.28	0.81^d^
**22.83**	**99.68**		5.56	4.82	3.06	4.58	2.75	0.97

*sensibility

** specificity

^d^ Non-differential misclassification

Under differential misclassification, we observed an inverse association moving away from the null as the sensitivity estimates were higher among cases than in controls, whereas when the controls had higher sensitivity estimates than cases we observed an increasing positive association pattern.

## Discussion

We hypothesized that Hypertensive Disorders of Pregnancy would be positively associated with cardiovascular diseases. After adjustment for smoking status, participant’s age, multiple gestations, family history of cardiovascular diseases, the total number of pregnancies, marital status and the level of education, we found a positive association between gestational hypertension and cardiovascular diseases, and an inverse association between preeclampsia and cardiovascular diseases.

The inverse association we found between preeclampsia and cardiovascular diseases, is counterintuitive; other studies found a positive association [5,7,15,16]. Our results may potentially be affected by recall bias and selection bias: Controls were much younger than cases (median age for controls was 29 vs 49 for cases), they were probably much better at recalling pregnancy outcomes than cases, since their pregnancies were more recent than cases’ pregnancies. Additionally, We assumed that pediatric consultations were not linked to pregnancy and Hypertensive Disorders of Pregnancy. However, In our study sample, preeclampsia, when treated as a dichotomous variable, had a similar distribution with Hypertensive Disorders of Pregnancy when treated as a dichotomous variable (both had slightly higher prevalences among controls than cases). The results from the sensitivity analysis on Hypertensive Disorders of Pregnancy suggested that, even if cases were more likely classified as exposed, the corrected estimates will be inverse and away from the null. This result suggests that a higher prevalence of the preeclampsia among the controls may have occurred if at the Gyneco-obstetric hospital of Yaoundé the files of neonate children were mixed with the files of children seeking for pediatric care. On the other hand, we cannot rule out the fact that children who were first admitted for neonatal care could have been readmitted for pediatric care. For these reasons, our initial assumption about independence between pediatric consultation and Hypertensive Disorders of Pregnancy may have not held, leading to a selection bias that increased the prevalence of preeclampsia among controls as Hypertensive Disorders of Pregnancy increase the likelihood of neonatal care. Also, some patients refused to participate, and others were deceased by the time of the study which may further increase the magnitude of selection bias.

The E-value for the association of Hypertensive Disorders of Pregnancy and Cardiovascular disease was as low as 1.70. This indicates that residual confounding may explain that we failed to find an association between both variables. We found evidence for the positive association between gestational hypertension in the polytomous model and weaker evidence for the same association in the dichotomous model, this could be explained by increased power due to the polytomous variable and a better adjustment for confounding, the E-value estimates were higher in the polytomous model than in the dichotomous model.

It has been demonstrated that questionnaires measuring Hypertensive Disorders of Pregnancy have low sensitivity and result in low powered studies [12,17]. This low sensitivity could explain that our study did not find any effect for gestational hypertension, and Hypertensive Disorders of Pregnancy when analyzed as dichotomous variables. Also, of concern, is the potential bias caused by participants who we could not reach and those who refused to participate.

Our results suggest that gestational hypertension is positively associated with cardiovascular diseases. Similar results were found by Ray et al.(2005) [16], Lykke et al. (2009) [18], Kestenbaum et al. (2003) [15], and Black et al.(2016) [19]. However, there is an open debate about the biological mechanism linking Hypertensive Disorders of Pregnancy to cardiovascular diseases later in life. Roberts and Hubel (2010) [4], stated that Hypertensive Disorders of Pregnancy did not cause cardiovascular diseases, rather they “Unravel” all the other risk factors of cardiovascular diseases. Hypertensive Disorders of Pregnancy and arteriosclerosis share endothelial dysfunction as a common characteristic. Some studies suggested that Hypertensive Disorders of Pregnancy are associated with unresolved endothelial dysfunction post-partum [20,21]. In fact higher levels of circulating anti-angiogenic factor sFlt1 and lower levels of circulating angiogenic factor, PIGF was found among women with previous Hypertensive Disorders of Pregnancy who had unresolved hypertension post-partum [22]. However, the exact period of endothelial dysfunction onset among women is uncertain. Noori et al. (2010) [23] observed that women who had lower flow-mediated dilatation in early pregnancy subsequently developed preeclampsia later. This suggests that endothelial dysfunction may happen even before pregnancy.

Women with a history of Hypertensive Disorders of Pregnancy also exhibit both impaired carbohydrate and lipid metabolism [24]. Hypertensive Disorders of Pregnancy have been demonstrated to increase insulin resistance [25]. Further, higher levels of very-low-density lipoprotein cholesterol (VLDL), as well as low-density lipoprotein (LDL), were found among women with a history of Hypertensive Disorders of Pregnancy [26,27]. Insulin resistance, as well as VLDL, and LDL may participate in endothelial dysfunction among women with Hypertensive Disorders of Pregnancy.

Our study has many limitations due to diverse forms of confounding and selection biases. We have not controlled for factors such as obesity. Low E-values estimates in the sensitivity analysis demonstrate our estimates are confounded. Selection bias appears in diverse forms in our study. First, in the form of Berkson’s bias as women of children admitted for neonatal care were included in the study. Mothers of children admitted to this service were more likely to have a diagnosis of Hypertensive Disorders of Pregnancy. Additionally, competing events and non-participation have also biased our estimates. Because of selection and confounding biases in our study, studies with a more accurate reporting of Hypertensive Disorders of Pregnancy and population-based samples are needed to provide unbiased and more precise estimates among African women.

However, we recommend that clinicians should screen and gather information about women past pregnancies. Roberts and Hubel (2010) [4] proposed a sample guideline for screening women about past pregnancies. Women who have experienced a Hypertensive Disorder of Pregnancy are also encouraged to frequently check their blood pressure and should adopt healthy lifestyles behaviours.

## Supporting information

S1 File Hypertensive disorders of pregnancy assessment questionnaire original. (DOCX)

S2 File Hypertensive disorders of pregnancy assessment questionnaire French. (DOCX)

S3 File Study data. (XLSX)

## References

[1] Abalos E, Cuesta C, Carroli G, Qureshi Z, Widmer M, Vogel JP, et al Pre‐eclampsia, eclampsia and adverse maternal and perinatal outcomes: a secondary analysis of the World Health Organization Multicountry Survey on Maternal and Newborn Health. BJOG: An International Journal of Obstetrics & Gynaecology. 2014; 121: 14–24. 10.1111/1471-0528.12629 24641531

[2] Abalos E, Cuesta C, Grosso AL, Chou D, Say L. Global and regional estimates of preeclampsia and eclampsia: a systematic review. Eur J Obstet Gynecol Reprod Biol. 2013; 170: 1–7. 10.1016/j.ejogrb.2013.05.005 23746796

[3] Williams D. Pregnancy: a stress test for life. Curr Opin Obstet Gynecol. 2003; 15: 465–471. 10.1097/00001703-200312000-00002 14624211

[4] Roberts JM, Hubel CA. Pregnancy. A screening test for later life cardiovascular disease. Women’s Health Issues. 2010; 20: 304–307. 10.1016/j.whi.2010.05.004 20800765

[5] Bhattacharya S, Prescott GJ, Iversen L, Campbell DM, Smith WC, Hannaford PC. Hypertensive disorders of pregnancy and future health and mortality: A record linkage study. Pregnancy Hypertens. 2012; 2: 1–7. 10.1016/j.preghy.2011.08.116 26104983

[6] Bellamy L, Casas JP, Hingorani AD, Williams DJ. Pre-eclampsia and risk of cardiovascular disease and cancer in later life: systematic review and meta-analysis. BMJ. 2007; 335: 974 10.1136/bmj.39335.385301.BE 17975258 PMC2072042

[7] McDonald SD, Malinowski A, Zhou Q, Yusuf S, Devereaux PJ. Cardiovascular sequelae of preeclampsia/eclampsia: a systematic review and meta-analyses. American heart journal. 2008; 156: 918–930. 10.1016/j.ahj.2008.06.042 19061708

[8] Lin Y-S, Tang C-H, Yang C-YC, Wu L-S, Hung S-T, Hwa H-L, et al Effect of pre-eclampsia–eclampsia on major cardiovascular events among peripartum women in Taiwan. The American journal of cardiology. 2011; 107: 325–330. 10.1016/j.amjcard.2010.08.073 21211611

[9] Diehl CL, Brost BC, Hogan MC, Elesber AA, Offord KP, TURNER ST, et al Preeclampsia as a risk factor for cardiovascular disease later in life: validation of a preeclampsia questionnaire. Am J Obstet Gynecol. 2008; 198: e11–3. 10.1016/j.ajog.2007.09.038 18241822

[10] Brown MC, Best KE, Pearce MS, Waugh J, Robson SC, Bell R. Cardiovascular disease risk in women with pre-eclampsia: systematic review and meta-analysis. European journal of epidemiology. 2013; 28: 1–19. 10.1007/s10654-013-9762-6 23397514

[11] Greenland S. Basic Methods for Sensitivity Analysis of Biases. Int J Epidemiol. 1996; 25: 1107–1116. 10.1093/ije/25.6.1107-a 9027513

[12] Klemmensen AK, Olsen SF, Osterdal ML, Tabor A. Validity of preeclampsia-related diagnoses recorded in a national hospital registry and in a postpartum interview of the women. American Journal of Epidemiology. 2007; 166: 117–124. 10.1093/aje/kwm139 17556761

[13] VanderWeele TJ, Ding P. Sensitivity analysis in observational research. introducing the E‐value. Annals of Internal Medicine. 2017; 167: 268–274. 10.7326/M16-2607 28693043

[14] Institut National de la Statistique, ICF International (2012) République du Cameroun Enquête Démographique et de Santé et à Indicateurs Multiples. Calverton, Maryland, U.S.A: ICF International

[15] Kestenbaum B, Seliger SL, Easterling TR, Gillen DL, Critchlow CW, Stehman-Breen CO, et al Cardiovascular and thromboembolic events following hypertensive pregnancy. Am J Kidney Dis. 2003; 42: 982–989. 10.1016/j.ajkd.2003.07.001 14582042

[16] Ray JG, Vermeulen MJ, Schull MJ, Redelmeier DA. Cardiovascular health after maternal placental syndromes (CHAMPS): population-based retrospective cohort study. The Lancet. 2005; 366: 1797–1803. 10.1016/S0140-6736(05)67726-4 16298217

[17] Valdiviezo C, Garovic VD, Ouyang P. Preeclampsia and hypertensive disease in pregnancy. Their contributions to cardiovascular risk. Clin Cardiol. 2012; 35: 160–165. 10.1002/clc.21965 22389120 PMC6652435

[18] Lykke JA, Langhoff-Roos J, Sibai BM, Funai EF, Triche EW, Paidas MJ. Hypertensive Pregnancy Disorders and Subsequent Cardiovascular Morbidity and Type 2 Diabetes Mellitus in the Mother. Hypertension. 2009; 53: 944–951. 10.1161/HYPERTENSIONAHA.109.130765 19433776

[19] Black MH, Zhou H, Sacks DA, Dublin S, Lawrence JM, Harrison TN, et al Hypertensive disorders first identified in pregnancy increase risk for incident prehypertension and hypertension in the year after delivery. J Hypertens. 2016; 34: 728–735. 10.1097/HJH.0000000000000855 26809018

[20] Agatisa PK, Ness RB, Roberts JM, Costantino JP, Kuller LH, McLaughlin MK. Impairment of endothelial function in women with a history of preeclampsia: an indicator of cardiovascular risk. American Journal of Physiology—Heart and Circulatory Physiology. 2004; 286: H1389–H1393. 10.1152/ajpheart.00298.2003 15020302

[21] Chambers JC, Fusi L, Malik IS, Haskard DO, de Swiet M, Kooner JS. Association of Maternal Endothelial Dysfunction With Preeclampsia. JAMA. 2001; 285: 1607–1612. 10.1001/jama.285.12.1607 11268269

[22] Goel A, Maski MR, Bajracharya S, Wenger JB, Zhang D, Salahuddin S, et al Epidemiology and Mechanisms of De Novo and Persistent Hypertension in the Postpartum Period. Circulation. 2015; 132: 1726–1733. 10.1161/CIRCULATIONAHA.115.015721 26416810 PMC4816491

[23] Noori M, Donald AE, Angelakopoulou A, Hingorani AD, Williams DJ. Prospective study of placental angiogenic factors and maternal vascular function before and after preeclampsia and gestational hypertension. Circulation. 2010; 122: 478–487. 10.1161/CIRCULATIONAHA.109.895458 20644016

[24] Paradisi G, Biaggi A, Savone R, Ianniello F, Tomei C, Caforio L, et al Cardiovascular risk factors in healthy women with previous gestational hypertension. J Clin Endocrinol Metab. 2006; 91: 1233–1238. 10.1210/jc.2005-1337 16434462

[25] Girouard J, Giguère Y, Moutquin J-M, Forest J-C. Previous Hypertensive Disease of Pregnancy Is Associated With Alterations of Markers of Insulin Resistance. Hypertension. 2007; 49: 1056–1062. 10.1161/HYPERTENSIONAHA.107.087528 17389257

[26] Sattar N, Bendomir A, Berry C, Shepherd J, Greer IA, Packard CJ. Lipoprotein subfraction concentrations in preeclampsia: pathogenic parallels to atherosclerosis. Obstetrics and gynecology. 1997; 89: 403–408. 10.1016/S0029-7844(96)00514-5 9052594

[27] Magnussen EB, Vatten LJ, Smith GD, Romundstad PR. Hypertensive disorders in pregnancy and subsequently measured cardiovascular risk factors. Obstetrics and gynecology. 2009; 114: 961–970. 10.1097/AOG.0b013e3181bb0dfc 20168095

