# Supplementary material for: Association between cardiovascular diseases and pregnancy-induced hypertensive disorders in a population of Cameroonian women at Yaoundé: A case-control study
Source: PLoS One. 2019 Dec 16;14(12):e0225591. doi: 10.1371/journal.pone.0225591 (PMC6913940; doi:10.1371/journal.pone.0225591)
Supplement: S1 File — (DOCX) [file pone.0225591.s001.docx]

**Project Title: Cardiovascular diseases risk in African women with history of hypertensive disorders of pregnancy who were patients at four first category hospital of yaounde**

**Project Dates:** July 2017 – December 2017

**Method:** Questionnaire

**Topic:** Cardiovascular diseases risk among African women with history of hypertensive disorders of pregnancy

**Target Audience:** Women who were patients at Yaoundé Central hospital, Yaounde Teaching hospital, Yaounde General hospital, and Yaoundé Gyneco-obstetric hospital

**Principal Investigator:**  Taa Nguimbis Esseme Benedict, BSc., MPH Candidate^1^ (benguimbis@live.fr).

Supervisor: Mbondji Ebongue Peter, PhD^1^ (mcbondj@yahoo.com)

1. Catholic University of Central Africa

# Instrument Title: History of Hypertensive disorders of pregnancy assessment Questionnaire

***Reserved to the investigator do not fill anything here***

|  |
| --- |

**ID Number**

|  |
| --- |

**Hospital**  1. Central Hospital 2. General hospital 3. University Hospital 4. Gyneco-obstetric hosp

|  |
| --- |

**Case situation**  1. Coronary heart disease 2. Cerebrovascular heart disease 3. Hypertension

# Start filling from here

**Inclusion Criteria: (*Answer the question by ticking the box beside the right answer or by filling the grey spots where appropriate)***

1. Are you from a sub-Saharan African Origin?

Yes: __________ No: ______________

1. . Have you had at least one pregnancy that lasted more than 5 months?

Yes: _________________________ No: _______________

1. Are you between 18 to 60 years old?

Yes: ________________________ No: ____________________

If you answered **NO** to any of the above questions, **STOP**. Please speak with a member of the Research staff immediately.

**Exclusion Criteria:**

1. Did you already have a cardiovascular disease or hypertension before your very first pregnancy?

Yes: ________________ No:_____________

1. Do you currently have any renal disease or Diabetes mellitus? Yes:________________ No:_____________ 3. Was your last pregnancy 6 months ago?

Yes: _____________ No: _______________

*If you answered* ***YES, STOP****. Please speak with a member of the Research Staff immediately.*

**Project Title: Cardiovascular diseases risk in African women with history of hypertensive disorders of pregnancy who were patients at four first category hospital of yaounde**

**Project Dates:** July 2017 – December 2017

**Method:** Questionnaire

**Topic:** Cardiovascular diseases risk among African women with history of hypertensive disorders of pregnancy

**Target Audience:** Women who were patients at Yaoundé Central hospital, Yaounde Teaching hospital, Yaounde General hospital, and Yaoundé Gyneco-obstetric hospital

**Principal Investigator:**  Taa Nguimbis Esseme Benedict, BSc., MPH Candidate^1^ (benguimbis@live.fr).

Supervisor: Mbondji Ebongue Peter, PhD^1^ (mcbondj@yahoo.com)

1. Catholic University of Central Africa

*Otherwise please continue onto the next page to fill out the questionnaire.*

We would like you to respond to the following questions. The questionnaire is meant to be anonymous your response will not allow us to identify you. The questionnaire will not be linked to your name. You do not have to answer every question.

## PART A: ASSESSMENT OF A DIAGNOSIS OF HYPERTENSIVE DISORDERS OF PREGNANCY

1. What is your year of birth? /_/_/_/_/(years)
2. . Have you had at least one pregnancy that lasted more than 5 months? 1.Yes __________

2. No _____________

1. During any of these pregnancies (which lasted more than 5 months), which of the following did the physician diagnosed? 1. Only high blood pressure or hypertension ___ 2. Pre-eclampsia

____ 3. Eclampsia ___ 4. None of these diseases ___

1. Was the physician diagnosis of hypertension or pre-eclampsia or eclampsia repeated in many of your pregnancies? 1.Yes ____ 2. No _____ 9. Do not know ___
2. If yes in how many pregnancies did a physician diagnose hypertension, pre-eclampsia or eclampsia? /_/_/_/

**PART B: ASSESSMENT OF CLINICAL MANIFESTATION OF HYPERTENSIVE DISORDERS OF**

## PREGNANCY

1. During any of your pregnancies (which lasted more than 5 months), did a physician ever tell you that you had high blood pressure or hypertension? 1.Yes __________ 2. No

_____________ 9. Do not know _____________

1. During any of your pregnancies (which lasted more than 5 months), were you prescribed any drug to lower your blood pressure (Adalate, Aldomet, Loxen, Tradate)? 1.Yes __________

2. No _____________ 9. Do not know _____________

1. During any of your pregnancies (which lasted more than 5 months), did a physician prescribed you aspirin, calcium supplements, or both? 1.Yes __________ 2. No _____________ 9.

Do not know _____________

1. During any of the pregnancies where you had a diagnosis of high blood pressure, or given drug to lower your blood pressure or aspirin (calcium supplement), did you have any convulsion or loss of consciousness? 1.Yes ______ 2. No ______ 9. Do not know _____
2. Did the high blood pressure and any of the symptoms disappear within the 03 months following delivery or the removal of the placenta? 1.Yes ____ 2. No ____ 9. Do not know ____

## PART C: COVARIATE ASSESSMENT

1. How many times have you already been pregnant? /_/_/_/
2. Have you already been pregnant of twins, triplets or more? 1.Yes __________ 2. No

_____________ 9. Do not know _____________

1. Which of the following describes you the best? 1.I smoke 2. I smoked but I quitted 3. I have never smoked

1. Do you have a relative (parents, grandparents, brothers, sisters, cousins, etc.) who has or had died from a cardiovascular disease or hypertension? 1.Yes __________ 2. No

_____________ 9. Do not know _____________

1. When was your last pregnancy (precise the year)? /_/_/_/_/________________
2. What is your educational level? 1.No level __________ 2. Primary _____________ 3.

Secondary _____________ 4. College/University_____________ 9. Do not Know

_____________

1. What is your marital status? 1. Single 2. Living with a companion but not married

3. Married 4. Divorced 5. Widow

1. Height (cm) of the participant /_/_/_/_/ Weight (kg) of the participant /_/_/_/_/ Waist circumference (cm) /_/_/_/_/ Hip circumference /_/_/_/_/
