# Supplementary material for: Association between cardiovascular diseases and pregnancy-induced hypertensive disorders in a population of Cameroonian women at Yaoundé: A case-control study
Source: PLoS One. 2019 Dec 16;14(12):e0225591. doi: 10.1371/journal.pone.0225591 (PMC6913940; doi:10.1371/journal.pone.0225591)
Supplement: S2 File — (DOCX) [file pone.0225591.s002.docx]

# Instrument Title: History of Hypertensive disorders of pregnancy assessment Questionnaire

***Reserve aux investigateurs ne rien écrire ici***

**ID de la participante: /_ /_ /_ /_ /_ /**

|  |
| --- |

**Hôpital**  1. Hôpital Central 2. Hôpital General 3. CHU 4. HGOPY

|  |
| --- |

**Case situation**  1. Maladie coronarienne 2. Maladie Cerebrovasculaire 3. Hypertension

4. Pas de maladies cardiovasculaires

Année du diagnostic ou du passage à l’hôpital /_ /_ /_ /_ /

**Répondez et remplissez à partir d’ici**

**Critères d’inclusion : (*Répondez aux questions en cochant à chaque fois la réponse la plus juste a pour chaque question)***

1. Etes-vous originaire d’Afrique sub-saharienne ?

Oui : __________ Non : ______________

1. Avez-vous déjà eu au moins une grossesse ayant dure au moins 5 mois ?

Oui : Non:

1. Avez-vous entre18 et 60 ans ?

Oui : ________________________ Non : ____________________

*Si vous avez répondu* ***NON*** *a au moins une des questions précédentes,* ***Arrêtez-vous****, et rapprochez-vous immédiatement d’un membre de l’équipe de recherche.*

**Critères exclusion :**

1. Avez-vous souffert d’hypertension ou de maladie cardiovasculaire avant votre toute première grossesse ?

Oui :________________ Non :_____________

1. Avez-vous une maladie rénale ou un diabète ?

Oui :________________ Non :_____________

1. Votre dernière grossesse remonte-t-elle a moins de 6 mois ?

Oui : _____________ Non : _______________

*Si vous avez répondu* ***OUI,*** *a au moins une des questions*  ***ARRETEZ VOUS****, et rapprochez-vous immédiatement d’un membre de l’équipe de recherche. Sinon continuez de remplir le questionnaire.*

Nous aimerions que vous répondiez à toutes les questions posées ici. Vos réponses seront anonymes et ne nous permettront pas de vous identifier. Les questionnaires ne seront pas lies à votre nom. Vous n’êtes pas obligées de répondre mais votre contribution en répondant a toutes les questions seront utiles.

***Cochez à chaque fois le carre à droite de la réponse qui décrit le mieux votre situation***

# PARTIE A : EVALUATION D’UN DIAGNOSTIC D’UNE MALADIE HYPERTENSIVE DE GROSSESSE

1. Quelle est votre année de naissance ? /_ /_ /_ /_ / (Années) (remplissez les cases)
2. Avez-vous déjà eu au moins une grossesse qui a duré au moins 5 mois ? 1.Oui 2.

Non

1. Durant l’une de vos grossesses, (qui a duré au moins 5 mois), laquelle des maladies suivantes votre médecin vous a diagnostiquée ? 1. Seulement la tension élevée ou hypertension 2.

Prééclampsies 3. Eclampsie 4. Aucune de ces maladies

1. Ce diagnostic de tension élevée (hypertension), ou de prééclampsies, ou encore d’éclampsies a-til été répété à certaines autres grossesses ? 1.Oui 2. Non 9. Je ne sais pas

## (si vous avez répondu autre chose que OUI passez directement à la partie B)

V. Si oui à combien de grossesses votre médecin vous a fait un diagnostic de tension élevée(hypertension), ou prééclampsies, ou encore éclampsies ? /_/_/ (remplissez les cases)

**PARTIE B : EVALUATION DES MANIFESTATIONS CLINIQUES DES MALADIES HYPERTENSIVES**

# DE LA GROSSESSE

1. Votre médecin vous a-t-il dit que vous souffriez de tension élevée ou d’hypertension durant ou pendant au moins une de vos grossesses qui a duré au moins 5 mois ? 1. Oui 2. Non 9. Je ne sais pas
2. Durant ou pendant au moins une de vos grossesses qui ont duré au moins 5 mois, votre médecin

vous a-t-il prescrit des médicaments (Adalate, Aldomet, Loxen, Tradate) pour traiter (soigner) votre tension ou hypertension ? 1.Oui 2. Non 9. Je ne sais pas

1. Durant ou pendant au moins une de vos grossesses qui ont duré au moins 5 mois, votre médecin

vous a-t-il prescrit de l’Aspirine ou des suppléments de Calcium pour votre tension élevée ou hypertension ? 1.Oui 2. Non 9. Je ne sais pas

*Si vous avez repondu* ***NON*** *a chacune des questions precedentes passez a la section C.*

1. Durant cette ou ces grossesses ou l’on vous a sois diagnostiquer une tension élevée, ou prescript des médicaments pour baisser votre tension, ou encore prescrit de l’aspirine et des suppléments de calcium, avez-vous eux des convulsions, des pertes de consciences, ou des évanouissements ?

1.Oui 2. Non 9. Je ne sais pas

1. Après votre accouchement, votre tension est-elle redevenue normale pendant les 03 mois qui ont suivi votre accouchement ? 1.Oui 2. Non 9. Je ne sais pas

# PARTIE C : EVALUATION DES COVARIABLES

1. Combien de fois avez-vous été enceintes ? /_/_ / (remplissez les cases)
2. Avez vous déjà été enceinte de jumeaux, triples ou plus ?

1.Oui 2. Non 9. Je ne sais pas

1. Laquelle de ces phrases est la plus juste vous concernant ? 1.Je fume 2. Je fumais, mais j’ai

arrêté 3. Je n’ai jamais fumé

1. Avez-vous un proche (frère, parent, oncle, tante, cousin, sœur) qui souffre ou qui sois mort de

suite d’une maladie cardiovasculaire ou du Cœur ? 1.Oui 2. Non 9. Je ne sais pas

1. En quelle année avez-vous accouché pour la dernière fois ? /_/_ /_/_/ (remplissez les cases)
2. Quel est votre niveau d’éducation ?

1.Je n’ai pas été à l’école 2. Primaire 3. Secondaire 4. Supérieur/Universitaire

9. Je ne sais pas

1. Quel est votre statut matrimonial ? 1. Célibataire 2. Je vis avec mon compagnon sans être

mariée 3. Mariée 4. Divorcée 5. Veuve

1. Taille (en cm) du participante /_/_/_/ Poids (en kg) de la participante /_/_ /_ / _/ Périmètre

abdominal(cm) /_/_ /_ /_/ Périmètre des Hanches(cm) /_/_/_/_/ (remplissez les cases)
